# An integrated computational framework to design a multi-epitopes vaccine against *Mycobacterium tuberculosis*

**DOI:** 10.1038/s41598-021-01283-6

**Published:** 2021-11-09

**Authors:** Aqel Albutti

**Affiliations:** Department of Medical Biotechnology, College of Applied Medical Sciences, Qassim University, Buraydah, Saudi Arabia

**Keywords:** Peptide vaccines, Adjuvants, Tuberculosis, Computational biophysics

## Abstract

Tuberculosis (TB) is a highly contagious disease that mostly affects the lungs and is caused by a bacterial pathogen, *Mycobacterium tuberculosis*. The associated mortality rate of TB is much higher compared to any other disease and the situation is more worrisome by the rapid emergence of drug resistant strains. Bacillus Calmette–Guerin (BCG) is the only licensed attenuated vaccine available for use in humans however, many countries have stopped its use as it fails to confer protective immunity. Therefore, urgent efforts are required to identify new and safe vaccine candidates that are not only provide high immune protection but also have broad spectrum applicability. Considering this, herein, I performed an extensive computational vaccine analysis to investigate 200 complete sequenced genomes of *M. tuberculosis* to identify core vaccine candidates that harbor safe, antigenic, non-toxic, and non-allergic epitopes. To overcome literature reported limitations of epitope-based vaccines, I carried out additional analysis by designing a multi-epitopes vaccine to achieve maximum protective immunity as well as to make experimental follow up studies easy by selecting a vaccine that can be easily analyzed because of its favorable physiochemical profile. Based on these analyses, I identified two potential vaccine proteins that fulfill all required vaccine properties. These two vaccine proteins are diacylglycerol acyltransferase and ESAT-6-like protein. Epitopes: DSGGYNANS from diacylglycerol acyltransferase and AGVQYSRAD, ADEEQQQAL, and VSRADEEQQ from ESAT-6-like protein were found to cover all necessary parameters and thus used in a multi-epitope vaccine construct. The designed vaccine is depicting a high binding affinity for different immune receptors and shows stable dynamics and rigorous van der Waals and electrostatic binding energies. The vaccine also simulates profound primary, secondary, tertiary immunoglobulin production as well as high interleukins and interferons count. In summary, the designed vaccine is ideal to be evaluated experimentally to decipher its real biological efficacy in controlling drug resistant infections of *M. tuberculosis*.

## Introduction

Tuberculosis or simply TB is an ancient disease and is of significant threat to world health for many years^1^. TB is ranked on top among infectious diseases and is associated with an increased number of deaths even in this advanced medical era^2^. The World Health Organization (WHO) reported 10 million TB cases and 1.2 million deaths in the year 2019. Each year, TB contributes to an estimated 1.5 billion infections resulting in 1.3 million deaths^3^. Also, WHO survey revealed 0.6 million cases of multidrug-resistant tuberculosis (MDR-TB) out of which 0.24 million infection leads to death^4^. Because of inappropriate use of antibiotics, the likelihood of emergence of new resistant TB phenotypes is high, warranting the development of new and safe TB vaccines^5^. More worrisome are the reports describing the impact of COVID-19 on TB management^6^. Recent studies indicated that COVID-19 could starch the TB mortality and morbidity level to that seen in the year 2015.

The use of Bacillus Calmette–Guérin (BCG) vaccine since 1923 is well established and is used globally^7^. Against adolescent pulmonary TB, the vaccine provides immune protection up to 80%^3^. The BCG vaccine is observed of low safety as the pathogen used in the vaccine has the potential of reactivation and risks of its use in immunocompromised people^8–10^. Many TB vaccines are in different phases of clinical trials. The viral vector-based vaccine like Crucell-Ad35/AERAS-402 and MVA85A have reduced protective efficacy^11^. The problem in using recombinant VPM1002 and MTBVAC is associated with the capability of returning to a virulent state^12^. The subunit vaccines like M72 and H4 comprise different mycobacterium antigens that lack strong immunogenicity, need multiple vaccinations, and need adjuvants^3^. One of the TB vaccines i.e. the M72/AS01 E was first considered safe for use in TB infected adults and healthy individuals but ruled out when several volunteers experienced local reactions at the site of injection during phase II trials^13^. With the success of peptide based vaccines including H4/IC31 due to its stable nature and accurate and specific immune responses, there is now much interest in developing a peptide vaccine for TB. Peptide vaccines are unveiled to be safe in phase I trials and disclosed to generate strong immune responses in BCG vaccinated infants and healthy adults^14^. Peptide vaccines are free from sequences that are associated with reactogenic responses and easy to produce^15^.

In the process to develop a more effective novel TB vaccine, several different approaches have been utilized. Among these, the development of a subunit vaccine attracted greater attention^16^. Subunit vaccines comprise a variety of epitopes and proved to be useful against TB based on data from clinical trials^17,18^. For example, Rodo et al. described that identification of potential subunit vaccine candidates which possess antigenic features have a higher possibility to be modeled into a safe vaccine candidate and could add valuable information for the production of an efficient TB vaccine^19^. Furthermore, epitope based vaccines are specific and accurate and can be easily produce^20^. Since cell immunity is proved to be more productive in protecting the host from TB infection, epitopes that stimulate both CD4^+^ and CD8^+^ T cells could be ideal to be considered in peptide based vaccine design^12^.

The present study aimed to propose a construct of multi-epitopes containing epitopes that are highly antigenic, non-allergenic, non-reactive to host immune system, and safe from toxic sequences. The prioritization of vaccine proteins was done from the core genome of 200 *Mycobacterium tuberculosis* species accessed on 2/7/2021. The core genome based vaccine designed ensured the selection of epitopes that are shared by all sequenced genomes of the pathogen, therefore, increase the chances of designing a broad-spectrum vaccine candidate. This approach also circumvents the limitation of several past computational studies where only one specific genome or weak methodology is utilized. The success of computational vaccines has been highlighted while identifying vaccine candidates against several infectious pathogens^21^ like Crimean-Congo hemorrhagic fever virus (CCHFV)^22^, Ebola virus^23^, severe acute respiratory syndrome coronavirus 2 (SARS-CoV-2)^24–26^, hepatitis C virus (HCV)^27^, schistosomes^28^, malaria^29^, *Acinetobacter baumannii*^30^, *Staphylococcus aureus*^31^, RSV^32^, and *Meningococcal meningitis*^33^*.* The selection of vaccine proteins was done considering proteins that are capable of activating cellular and humoral immunity. The finding from the current study will not only add novel peptide vaccine candidates against TB but also can be applied in experimental research in an effort to eradicate TB.

## Materials and methods

**Figure 1:**
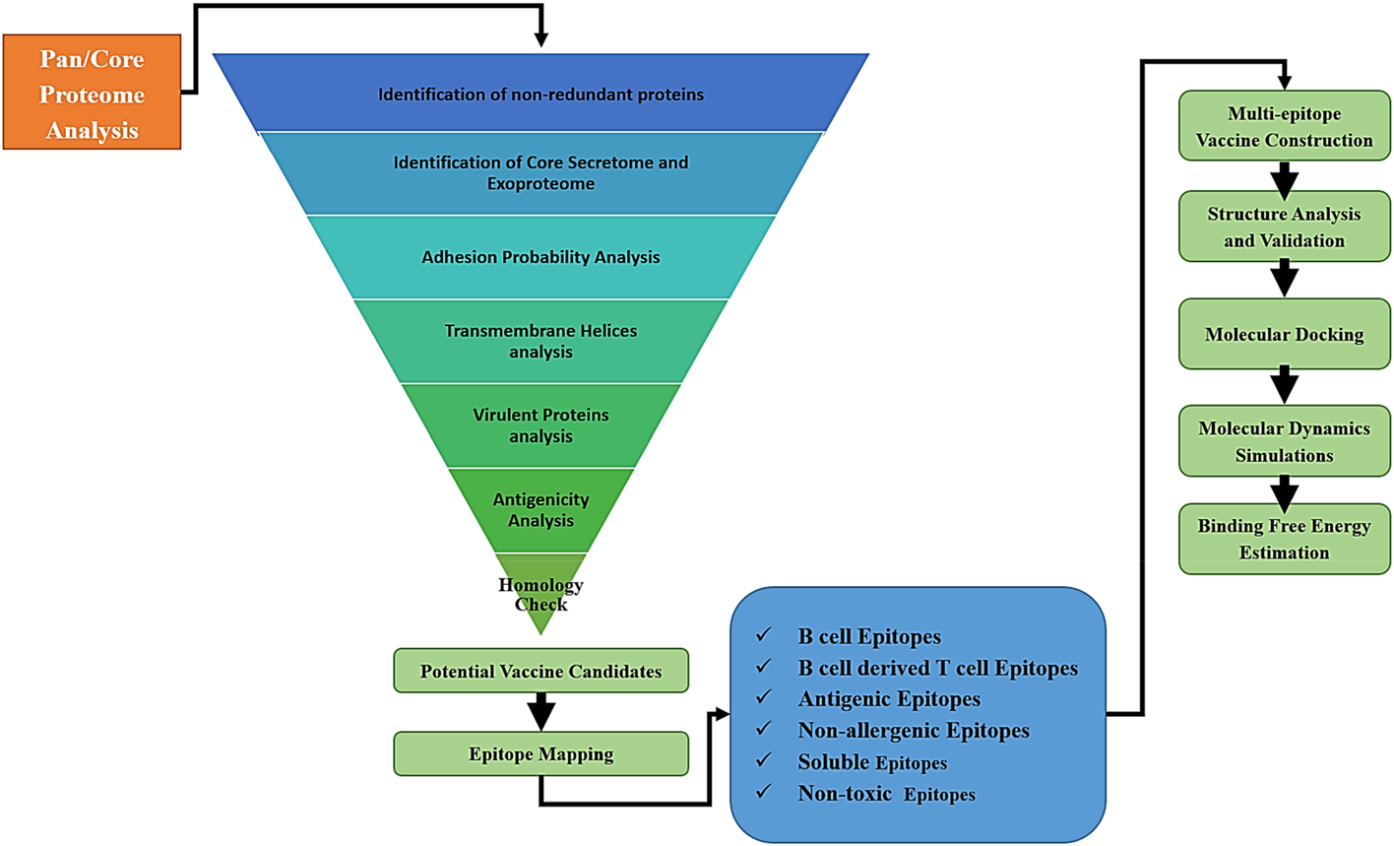
Schematic flow of the work performed in this study.

The workflow of the present study is schematically shown in Fig. 1.

### Collection of genomics data

Genomic data of all available strains of *M. tuberculosis* were retrieved from the genome database of the national center for biotechnological information (NCBI)^34^. Additional information of each strain like name, accession number, GC%, and the number of contigs were also retrieved.

### Pan-genomics analysis

Bacterial pan-genome analysis (BPGA)^35^ tool was employed to perform pan-genomic analysis of 200 fully sequenced genomes of the selected pathogen. First, 90% sequence identity cut-off value is considered for clustering of proteome sequences in the USEARCH algorithm. The graphs for core and pan genomes were generated by investigating the total number of shared and unique gene families against the total set of genes. Moreover, BPGA output includes gene family distribution plots describing core pan and accessory proteins, distribution plots of new genes added with the addition of each successive genome. Evolutionary analysis was done in BPGA considering concatenated core gene alignments and binary (presence/absence) pan-matrix. The gene matrix is generated based on the similarity/dissimilarity of genes in orthologous gene clusters. In the core genome phylogenetic tree, orthologous gene sequences were extracted from 20 random clusters, followed by multi-locus sequence typing (MLST) analysis on selected housekeeping genes. The alignments were then concatenated and a neighbor-joining phylogenetic tree was constructed. The core genome sequences of *M. tuberculosis* obtained through BPGA were subjected to literature reported vaccine filters to identify potential vaccine targets.

### Identification of non-redundant genes

The core genome was then analyzed for the presence of redundant genes. Redundant genes are duplicate sequences and are the product of duplication events during the evolution process. As such duplicate sequences are not required and only a single representation of each gene is needed, a CD-Hit check was performed to remove redundancy from the core sequence^36,37^. For this purpose, CD-Hit Suite^37^ was employed and the core genome was filtered in three consecutive steps: first at 90% sequence similarity, followed by 60% and 50%. The non-redundent genes were translated into proteins using the Prokaryotic dynamic programming gene finding algorithm (Prodigal)^38^.

### Prioritization of potential vaccine candidates

The core proteome non-redundant sequences were analyzed in subcellular localization, which was performed via PSORTb v3.0.2^39^ and cross-validated by CELLO v.2.5^40^. Only proteins localized at the outer surface or secretory in nature were opted. Surface localized proteins not only interact with host cells but also due to their exposed nature and harboring antigenic determinants they are considered are good candidates to stimulate the human immune system and generated rigorous immune responses^41^. Next, adhesion potential of the shortlisted proteins was examined using Vaxign^42^ with a threshold set to 0.5. Adhesive proteins facilitate bacterial attachment to the host cells and are regarded as good vaccine candidates^43,44^. These proteins generate specific antibodies response and activate protective immunity^43^. Both outer surface and adhesive proteins are considered good vaccine candidates as they are highly antigenic and because of their exposed nature, they can be easily recognized by host immune cells^45^. Moreover, the number of transmembrane helices for each adhesive protein were predicted using HMMTOP^46^ to ensure easy laboratory analysis of selected vaccine candidates. Less number of transmembrane helices ensured easy purification, cloning and expression analysis^47^. Virulent proteins were identified through the use of virulent factor database (VFDB)^48^ considering selection criteria of > 30% sequence identity and bit score > 100. Virulent proteins are regarded as good vaccine targets as they have the potential to stimulate infection and immune system pathways^41^. VaxiJen 2.0 server^49^ was used to analyze the antigenicity of the filtered proteins obtained in the previous step. During this analysis, the cut-off score was adjusted to 0.5. The prediction accuracy of this server is between 70 to 89% range. The good accuracy of this tool neglects the chances of selecting false positive hits. Determining antigenicity is essential as those proteins can be selected which can generate good antibody responses^50^. Next, human homologous, as well as mice and pig homologous proteins, were excluded from antigenic proteins^41^. This check was important to evaluate as significant sequence similarity with the mentioned organisms can either cause autoimmunity reactions or tolerance to the vaccine antigen. This in turn could lead to harmful consequences on the host’s health. Hence, bacterial proteins showing homology to the hosts proteome are not considered safe vaccine candidates^45^, therefore not processed further in the pipeline.

### Identification of B-cell epitopes

B-cell epitopes in the selected set of proteins were used for prediction of B-cell epitopes using immune epitopes database (IEDB)^51^ and those with a prediction score of > 0.5 were considered in the next step.

### Identification of cytotoxic T lymphocytes epitopes

The cytotoxic T lymphocytes epitopes were predicted using NetCTL 1.2 server^52^. The epitopes prediction is done based on three main parameters including an affinity for MHC-I binding, artificial neural networks (ANN) based on proteasomal C terminal cleavage, and transporter associated with antigen processing (TAP). These epitopes prediction was done by setting a different value for each of the above parameters. For example, TAP transport efficiency cut-off value is set to 0.05, proteasomal C-terminal cleavage value is 0.15, and epitope identification is 0.75. The predicted epitopes were ordered based on a combined score of the set parameters.

### Identification of helper T lymphocytes epitope

To predict helper T lymphocytes epitopes, the IEDB MHC II server^51^ was employed. Human/HLA-DR was chosen as the species/locus and a 7-allele human leukocyte antigen (HLA) reference set was considered for the HTL epitopes prediction. The epitopes length was set to 15-mer and ranked as per percentile score. The percentile rank is assigned to the epitopes after comparing the epitope score with 5 million 15-mer from the SWISSPROT database. The lowest percentile score represents a high affinity of the epitopes for MHC-II alleles.

### Construction of multi-epitopes subunit vaccine

For design of a multi-epitopes construct that has the potential to stimulate both innate and adaptive immune responses, the best rank helper and cytotoxic epitopes screened above were selected. Epitope joining was done using AAY (between cytotoxic epitopes) and GPGPG linkers (between helper epitopes) that were added between the epitopes. A TLR-4 agonist (RS-09; Sequence: APPHALS) was added to the vaccine construct as an adjuvant using EAAAK linker. RS09 enables stimulation of T-cells receptors and driving a more robust immune activation. The use of RS09 as a synthetic adjuvant is a safer approach and regarded as more advance than traditional Freund’s adjuvant^53^. The construct 3D structure was modeled using ab initio method through 3Dpro server^54^. Ab initio modelling was done as no appropriate template for the vaccine structure modelling was available^55^. The structure was then subjected to loop modeling using Galaxyloop^56^ and refined via GalaxyRefine^57^.

### Physicochemical features characterization

To characterize physicochemical features of selected proteins, Protparam tool^58^ was used. Physicochemical analysis was important to guide experimentalist in in vitro and in vivo analysis of the designed vaccine construct^59^. Several different properties of the vaccine were evaluated. Molecular weight of the vaccine was examined and selected if the size is smaller than 110 kDa^41^. Small size proteins are easy to clone and express in expression systems compared to large size proteins^31^. Aliphatic index and hydrophobicity assays were done to evaluate thermally stability and hydrophilic nature of the vaccine. The GRAVY index in negative is an indication of hydrophilicity whereas a greater aliphatic index (> 50) implies good stability at varying temperatures.

### Molecular docking studies

Molecular docking is a highly useful technique to predict the preferred binding mode of a ligand molecule with respect to its receptor^60^. Herein, the vaccine ability to interact with appropriate immune receptors (TLR4, MHC-I and MHC-II) was evaluated through a molecular docking approach. The PDB files of the mentioned receptors were first obtained from protein data bank (pdb)^61^ using the following codes; TLR4 (PDB ID: 4G8A), MHCI (PDB ID: 1I1Y) and MHCII (PDB ID: 1KG0). The docking was blindly performed using a PATCHDOCK server^62^. Prior to docking studies, the vaccine and receptor molecules were prepared in UCSF Chimera 1.13.1^63^ and energy minimized for 750 steps of conjugate gradient and steepest gradient. During the docking procedure, the clustering RMSD is set to 4.0 Å. The docked solutions were further refined through Fast Interaction Refinement in Molecular Docking (FireDock) server^64^. The server remove high energy contacts from complexes and rank top the solution with best energy minima. The selection of docked complex for simulation studies was based on the lowest global energy. UCSF Chimera 1.13.1 was used for all binding modes and interactions visualization.

### Molecular dynamics simulations

The dynamic behavior of vaccine-receptors complex was studied to determine the strength of intermolecular interactions verses time as well as to check whether the vaccine epitopes are exposed to the host immune system for recognition and efficient processing^30,65^. The selected docked complexes were subjected further to 200 ns of molecular dynamics simulation performed through AMBER20 simulation package^66^. The procedure was applied in three phases: preparation of parameter files, pre-processing to make the complex files ready for simulation production run, and production for structure stability analysis^67,68^. The antechamber^69^ of the AMBER was applied to generate complex libraries and initial parameters. The complex was submerged into 12 Å TIP3P water box using Leap module. The ff14SB force filed^70^ was used for treating the complex and counter ions were added to neutralize the system. System energy minimization was performed for hydrogen atoms, water box, complete atoms and non-heavy atoms energy minimization. Heating of systems was achieved through NVT ensemble and the temperature was maintained at 300 K. SHAKE algorithm^71^ was used to restrain hydrogen bonds. The system was then equilibrated for 100 ps. Pressure on the system was maintained using NPT ensemble while applying restrain on carbon alpha atoms. The simulation production was run for 200 ns and trajectories were produced on time scale of 2 fs. The simulation trajectories were analyzed through AMBER CPPTRAJ module^72^ and structure stability plots were generated via Xmgrace software^73^.

### Binding free energy estimation

Estimation of vaccine ensemble binding free energy to the immune receptors was carried out to validate binding stability of the docked vaccine-immune receptors complex. This was done using molecular mechanics generalized Poisson Boltzmann surface area (MMPBSA) method^74^ of AMBER20. This binding energy method is robust, reliable, and widely employed because of its modest running requirements. The MMPBSA.py script^75^ of the AMBER was utilized to estimate net binding free energy over 500 selected frames of simulation trajectories. The following equation is used to calculate net binding free energy,

$$\Delta Gbinding=\Delta EMolecularMechanics+\Delta Gsolvation-T\Delta G\Delta Gbinding=\Delta EMolecularMechanics+\Delta Gsolvation-T\Delta S(entropy)$$

### Immune simulation studies

Online server of C-ImmSim, server^76^ (http://150.146.2.1/C-IMMSIM/index.php) was used to evaluate the possible host immune responses against the vaccine antigen. The server deciphers both cellular and humoral immune responses against the vaccine. The designed vaccine antigen was administered in the form of three injections at an interval gap of 4 weeks (1, 84, and 168). The simulation volume, number of simulation steps, and number of seeds employed are at 10, 1000, 12,345, respectively. Sulfatide (id: 135,039,462) was used as an adjuvant molecule^77,78^. A recent study has shown sulfatide as a promising adjuvant in an oral cholera vaccine^79^ therefore we are expecting to use the sulfatide in oral vaccine design against *M. tuberculosis*.

### Codon adaptation and in silico cloning

To optimize the high expression of the vaccine in an appropriate expression vector, the constructed vaccine sequence was used as input into the Java Codon Adaptation Tool (JCAT)^80^ for codon optimization. The *Escherichia coli* strain K12 was set as a host system for expressing the vaccine. In the process, three different options were selected in the JCAT server. These include avoiding prokaryotes ribosome binding sites, rho-independent transcription, and restriction enzymes cleavage sites. The expression rate was determined through parameters like codon adaptation Index and GC content. The restriction cloning module of the SnapGene tool was also utilized to clone the vaccine sequence into *E. coli* pET-28(+) vector.

### Disulfide engineering for vaccine stability

Further, the vaccine stability was increased by adding disulfide bonds replacing highly unstable residues in the vaccine ensemble. The Disulfide by Design v2.0 server^81^ was used to perform disulfide engineering of the vaccine construct. The vaccine model was initially run to identify residue pairs to be used in disulfide engineering. Residue pairs with net energy higher than 3 kcal/mol were opted for mutation. In total, 8 residue pairs were considered for mutation by Disulfide by Design server.

## Results and discussion

The results of the current work can be discussed under the following headings.

### Pan-genome analysis of *M. tuberculosis*

**Figure 2:**
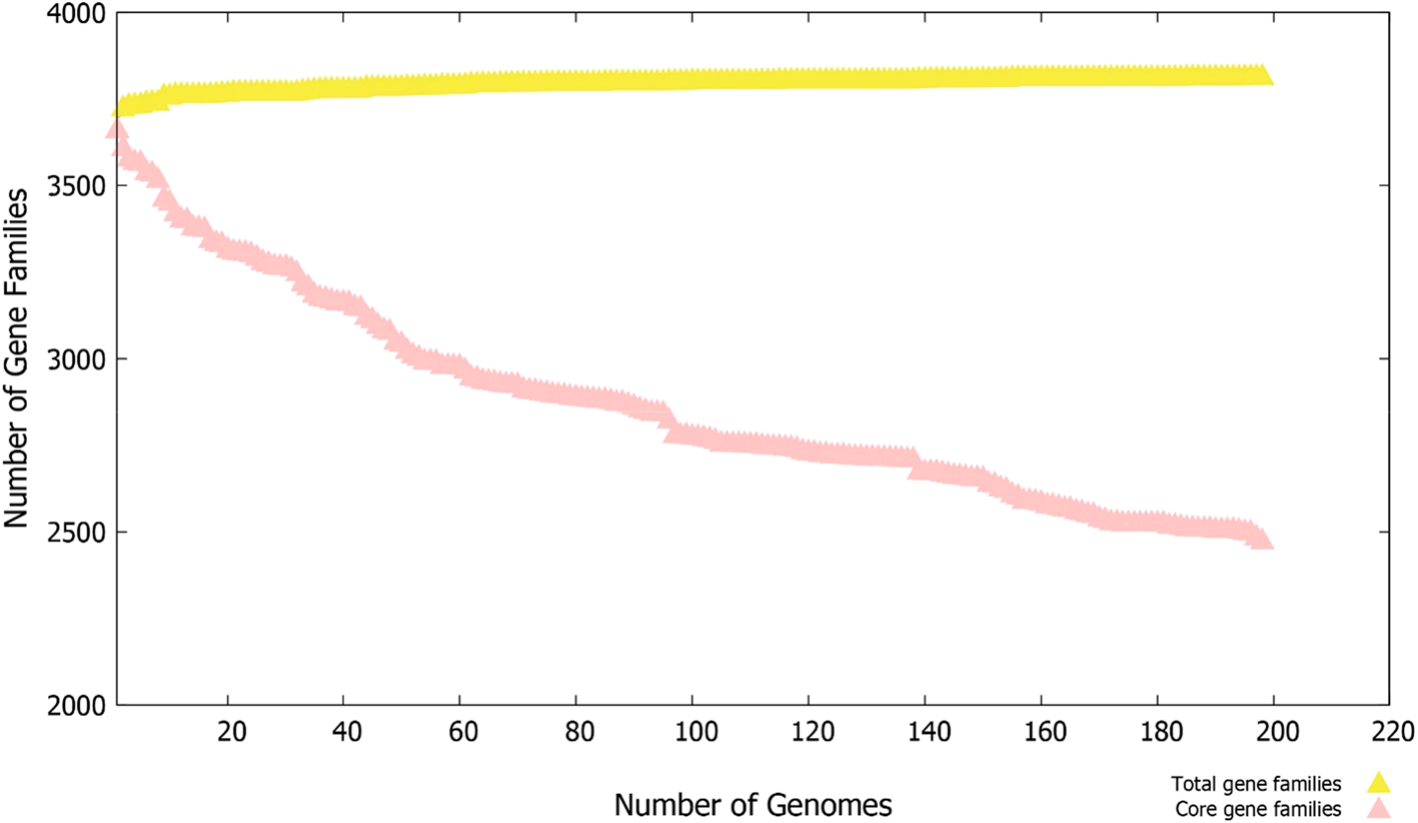
Pan-genome anlaysis plot of 200 *M. tuberculosis genomes*.

The fully sequenced 200 genomes of *M. tuberculosis* were retrieved from Genome database of NCBI that on average contain 3890 proteins per strain and a total of 906,505 proteins. The different information of genomes used in the analysis is tabulated in Table S1. The total number of genes clustered for pan-genome is 766,485 while pan-genomic analysis found 490,248 genes that constitute the core genome of *M. tuberculosis.* The average number of core genes, accessory genes, unique genes, and exclusively absent genes are 2476, 1198, 1, and 3, respectively. The contrast between the total and core gene families is shown in Fig. 2 which demonstrates the open state of pan-genome and the addition of new genes in the future that will affect the pathogen pan-genome. Further, Clusters of Orthologous Groups (COG) distribution analysis unraveled that majority of the core genes are involved in metabolic biogenesis and regulation. On the other side, unique genes function in storage and information processing which encode different classes of proteins and can be subdivided into replication proteins, RNA processing proteins, recombination proteins, transcription/ translation proteins, and chromosomal dynamics proteins. The number of core, accessory, unique and absent genes in each strain is presented in Table S2. Additionally, the core genome and pan-genome phylogenetic trees were generated and analyzed as presented in Figs. S1 and S2. The core sequences based tree is obtained based on concatenated genes alignment of the core genome while pan-phylogeny is generated based on accessory gene presence/absence in different strains of *M. tuberculosis*. Variations can be seen in the phylogenetic relationship among the strains as evident by different clustering and placements of strains in the clusters.

### Reverse vaccinology analysis

Reverse vaccinology approach was used to prioritize potential vaccine candidates from the core sequences based on serval vaccine properties. The number of proteins along each of the reverse vaccinology framework is tabulated in Table 1.

**Table 1:** Different properties of filtered exo-proteome and secretome of the *M. tuberculosis* helped in identify potential candidates for epitope mapping.

Protein Accession	Localization	Adhesion Probability	Trans-membrane HELICES	VFDB		Antigenicity	Homology Analysis	Probiotics Analysis		
				Bit Score	Sequence Identity			*L. rhamnosus* 47,715	*L. casei* 1582	*L. johnsonii* 33,959)
core/1445/1/Org1_Gene111	Extracellular	0.812	1	629	92%	0.4472(Antigen)	Non Similar	Non similar	Non similar	Non similar
core/1852/1/Org1_Gene3213	Extracellular	0.305	0	95.9	48%	0.5303(Antigen)	N.D	N.D	N.D	N.D
core/2239/1/Org1_Gene1513	Extracellular	0.49	0	33.1	32%	N.D	N.D	N.D	N.D	N.D
core/2346/1/Org1_Gene1193	Extracellular	0.749	1	32.3	39%	N.D	N.D	N.D	N.D	N.D
core/2752/1/Org1_Gene1149	Extracellular	0.592	0	33.9	27%	N.D	N.D	N.D	N.D	N.D
core/2768/1/Org1_Gene1723	Extracellular	0.654	1	35	28%	N.D	N.D	N.D	N.D	N.D
core/2813/1/Org1_Gene3233	Extracellular	0.752	0	35.8	33%	N.D	N.D	N.D	N.D	N.D
core/3428/1/Org1_Gene19	Extracellular	0.574	0	157	85%	0.8036 (Antigen)	Non-Similar	Non-similar	Non-similar	Non-similar
core/3438/2/Org2_Gene1116	Extracellular	0.701	0	82.4	57%	N.D	N.D	N.D	N.D	N.D
core/1269/3/Org3_Gene2358	Extracellular	0.886	0	31.6	23%	N.D	N.D	N.D	N.D	N.D
core/13/4/Org4_Gene3837	Extracellular	0.924	0	89	42	N.D	N.D	N.D	N.D	N.D
core/293/33/Org33_Gene3334	Extracellular	0.798	0	51.2	46%	N.D	N.D	N.D	N.D	N.D
core/13/52/Org52_Gene3827	Extracellular	0.89	0	89	42%	N.D	N.D	N.D	N.D	N.D

If a protein fails in one of the given analysis that protein was not forwarded to the next analysis.

*N.D* not done.

#### Identification of non-redundant proteins

The core genome sequences obtained in the previous step were then subjected to a redundancy check to remove duplicate copies. This ensures that only one copy of each gene is present in the core sequence file thus reducing computational efforts and only analyze relevant genes for potential vaccine candidate’s identification^36^. After this analysis, the number of non-redundant and core genes achieved is reduced to 2598. These non-redundant genes are orthologs and play a significant role in the context of vaccine design.

#### Identification of core secretome and exoproteome

Next, the non-redundant core proteome was subjected to subcellular localization analysis to opt for proteins that form the pathogen secretome and exoproteome. These proteins directly interact with the host immune system and harbor antigenic determinants capable of eliciting strong immune responses^47^. Also, these proteins function as virulent, adhesive, invasive, and proliferators. These properties make such proteins good targets for the identification of antigenic epitopes which can be used in making a chimeric vaccine construct. Subcellular localization analysis identified 14 extracellular/outer membrane proteins while 2,584 were either cytoplasmic, cytoplasmic membrane, or of nuclear origin.

#### Adhesion probability analysis

The screened exoproteome and secretome were further investigated for adhesion probability to filter only those proteins that can bind to the host cells and mediate infection pathways. Adhesive proteins can be easily recognized by antibodies are they are major players in initial colonization and pathogenesis^44^. It has been reported that FimH adhesive protein of *Escherichia coli* is capable of stimulating antibodies production to impede bacterial adherence^82^. Only one protein was found non-adhesive, while 13 proteins were characterized as adhesive and were passed to the next check.

#### Transmembrane helices analysis

The number of transmembrane helices for each shortlisted protein was then evaluated to ensure selection of those proteins which can be easily purified, clone, and express in follow up experimentations^41^. Usually, transmembrane helices should be < 1. All the 13 protein were reported to have either 1 or 0 transmembrane helices, therefore, were considered for downward analysis.

#### Virulent proteins analysis

Virulent proteins are important mediators and help in activating the host immune system in response to the vaccine antigen^83^. According to the selection criteria described in the methodology section, two proteins were selected as virulent. The bit score and sequence identity score of both these proteins are > 100 and 30%, respectively. These proteins are diacylglycerol acyltransferase and ESAT-6-like protein. Diglyceride acyltransferase aids in the formation of triglycerides from Acyl-CoA and diacylglycerol and is considered the terminal step in triglyceride synthesis^84^. ESAT-6-like protein is a secretory antigen protein of *M. tuberculosis*^85^.

#### Antigenicity analysis

Both selected proteins were further examined for antigenicity. Antigenicity refers to the potential of antigen to bind antibodies and T cell receptors. Both the proteins were disclosed to have strong antigenic properties because they are localized at the pathogen surface and contain antigenic sequences. Antigenic proteins form the foundation of vaccine design and thus both proteins were analyzed for additional analysis.

#### Homology check

Next, selected diacylglycerol acyltransferase and ESAT-6-like proteins were subjected to homology check against Homo sapiens reference proteome as well as that of mouse and pigs. Both the proteins were found to have less homology than the set criteria: (sequence identify < 30%, bit score < 100, and E-value < 1.0 E^−5^). This check was necessary as homologous proteins with the host proteome can lead to autoimmune reactions and can cause immune reactions against self-antigens^86^. The non-homologous nature of the above mentioned proteins to the mouse and pigs also is important as it can help in negating false-positive results during in vivo experiments^42^.

#### Comparative homology analysis with probiotic bacteria

**Figure 3:**
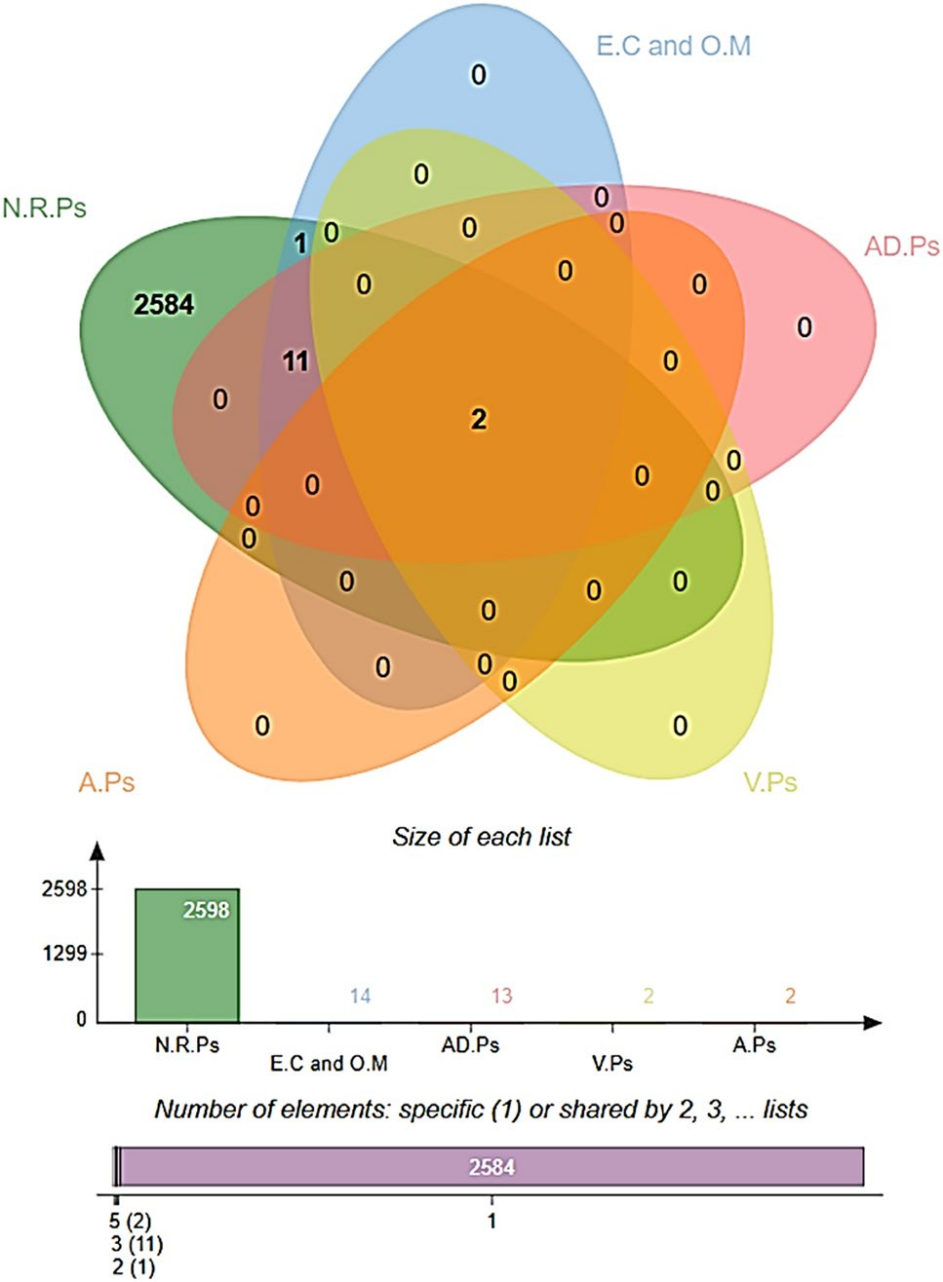
Prioritization of potential vaccine candidates using reverse vaccinology approach. The proteins selection is based on different vaccine parameters. N.R.Ps (non-redundant proteins), E.C (extracellular), O.M (outer membrane), AD.Ps (adhesive proteins), V.Ps (virulent proteins) and A.Ps (antigenic proteins).

To make sure that no damage is conferred to the host beneficial probiotic bacteria, a homology BLASTp check was also carried out against three reference species of *Lactobacillus* species; *L. rhamnosus* (taxid: 47715), *Lactobacillus casei* (taxid: 1582), and *L. johnsonii* (taxid: 33959)^87^. The proteins were unveiled to have no significant hits against the search species and were considered as non-homologous to the probiotic bacteria. The number of proteins gets at each step of reverse vaccinology is presented in the form of a Venn diagram (Fig. 3).

### Epitome analysis

**Figure 4:**
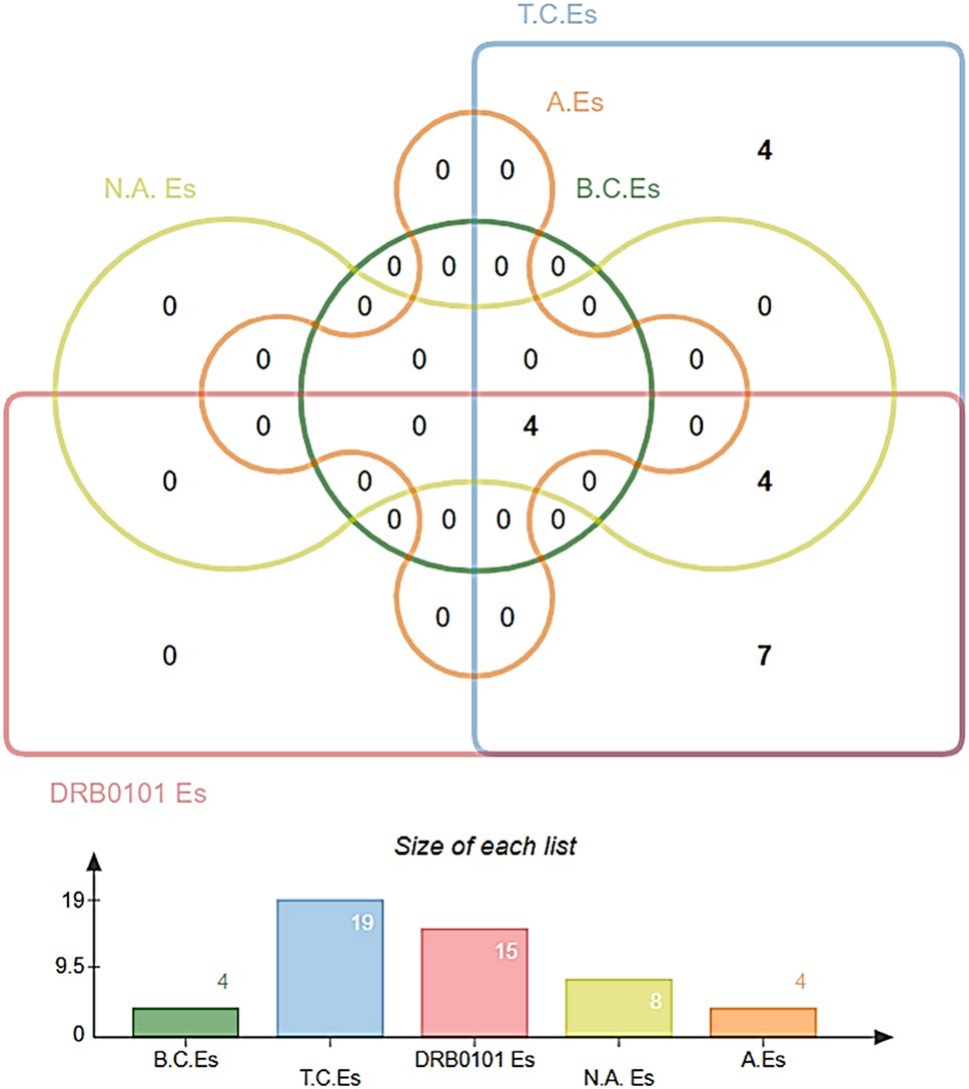
Number of epitopes achieved at each step of epitope mapping phase.

The two potential vaccine proteins: diacylglycerol acyltransferase and ESAT-6-like proteins fulfilled all the vaccine properties and were thus considered for epitome analysis while the rest of the proteins were discarded from the pipeline. Epitopes identification is vital for many reasons including immune monitoring, devising new diagnostics assays, and epitopes based vaccine design^88^. In the first step of this phase, B-cell epitopes were predicted for the proteins. A B-cell epitope refers to an antigen site that binds to antibodies or immunoglobulin. B-cells epitopes prediction found three epitopes for diacylglycerol acyltransferase and one epitope for ESAT-6-like protein. The T-cell epitopes were predicted next. T-cell epitopes can stimulate either CD4 helper or CD8 cytotoxic T-cells. For diacylglycerol acyltransferase, six MHC-II epitopes were predicted while 13 MHC-I epitopes were found to be harbored by MHC-II. In case of ESAT-6-like protein, two MHC-II and sic MHC-I epitopes were predicted. A cross-check analysis was then accomplished to select only those peptides that are presented in both B-cell and T-cell epitopes. The epitopes were forwarded to MHCpred analysis to highlight epitopes that have a higher affinity for the highly prevalent DRB1*0101 allele in the human population^45,89^. Epitopes that show good binding with the said allele lead to good immune responses. Only DRB1*0101 allele binders that have an IC50 value less than 100 nM were selected as they represent strong binders. 15 epitopes were screened as strong DRB1*0101 allele binders. Next, the filtered epitopes were validated for allergenicity and antigenicity to avoid the selection of allergic epitopes and non-antigenic epitopes. At the end of this phase, solubility and toxicity of the epitopes were examined. Only, soluble and non-toxic epitopes were considered for multi-epitopes vaccine construction. In a nutshell, four epitopes (DSGGYNANS from diacylglycerol acyltransferase and AGVQYSRAD, ADEEQQQAL, and VSRADEEQQ from ESAT-6-like protein) were found as good epitopes as they are B-cell derived T-cell epitopes, have the good binding potential for DRB1*0101 allele, non-allergens, antigenic, soluble in water and are non-toxic. The different epitopes analysis performed are described in Table 2 while the number of epitopes gets at each step of epitope mapping phase is presented in Fig. 4.

**Table 2:** Epitope mapping analysis for potential vaccine candidates.

Sequence	B—cell epitopes	MHC-II	Percentile Score	MHC-I	Percentile Score	MHCPred (DRB0101 binder	IC50 Score	Allergenicity	Antigenicity	Water Solubality Analysis	Toxicity Analysis
core/1445/1/Org1_Gene111	GTFGGPATAGAFSRPGL	GTFGGPATAGAFS	0.9	GTFGGPATA	0.22	GTFGGPATA	1452.11	Allergens	− 0.4485	N.D	N.D
				GPATAGAFS	3.2	GPATAGAFS	833.68	Non-allergen	0.3567	N.D	N.D
		GPATAGAFSRPGL	6.7	GPATAGAFSR	1.8	PATAGAFSR	3.99		N.D	N.D	N.D
						GPATAGAFS	833.68	Non-allergen	0.3567	N.D	N.D
				AGAFSRPGL	1.8	AGAFSRPGL	3459.39	Non-allergen	− 0.5343	N.D	N.D
	FYTDWYQPSQSNGQNYTYK	TDWYQPSQSNGQN	5.8	YQPSQSNGQ	8.6	YQPSQSNGQ	12.71	N.D	N.D	N.D	N.D
						TDWYQPSQS	32.43	N.D	N.D	N.D	N.D
				TDWYQPSQSN	20	DWYQPSQSN	169.43	Allergens	N.D	N.D	N.D
		YQPSQSNGQNYTY	21	SQSNGQNYTY	0.02	SQSNGQNYT	61.38		N.D	N.D	N.D
						QSNGQNYTY	642.69	Allergens	N.D	N.D	N.D
				YQPSQSNGQ	8.6	YQPSQSNGQ	12.71	N.D	N.D		
	DSGGYNANSMWGPSSDPAWKR	DSGGYNANSMWGP	22	GGYNANSMW	0.39	GGYNANSMW	335.74	Allergens	N.D	N.D	N.D
				DSGGYNANSM	5.8	SGGYNANSM	47.75	N.D	N.D	N.D	N.D
						DSGGYNANS	252.93	Non-allergen	1.5288	Good water solubility	Non-toxic
				YNANSMWGP	16	YNANSMWGP	117.49	Non-allergen	− 0.0945	N.D	N.D
		SMWGPSSDPAWKR	39	GPSSDPAWKR	0.81	PSSDPAWKR	3.3	N.D	N.D	N.D	N.D
						GPSSDPAWK	277.97	Allergens	N.D	N.D	N.D
				SMWGPSSDPA	1.8	SMWGPSSDP	34.83	N.D	N.D	N.D	N.D
						MWGPSSDPA	39.63	N.D	N.D	N.D	N.D
> core/3428/1/Org1_Gene19	RQAGVQYSRADEEQQQALSS	AGVQYSRADEEQQ	5.9	VQYSRADEE	5.9	VQYSRADEE	101.86	Allergens	N.D	N.D	N.D
				AGVQYSRADE	48	GVQYSRADE	108.89	Allergens	N.D	N.D	N.D
						AGVQYSRAD	389.94	Non-allergen	0.8762	Good water solubility	Non-toxic
				YSRADEEQQ	10	YSRADEEQQ	20.7	N.D	N.D	N.D	N.D
		SRADEEQQQALSS	39	ADEEQQQAL	0.66	ADEEQQQAL	120.5	Non-allergen	0.8792	Good water solubility	Non-toxic
				EEQQQALSS	1.4	EEQQQALSS	4.05	N.D	N.D	N.D	N.D
				VSRADEEQQ	9.7	VSRADEEQQ	743.02	Non-allergen	1.0484	Good water solubility	Non-toxic

If a epitope fail in one of the given analysis that epitope was not forwarded to next analysis.

*N.D* not done.

### Multi-epitopes peptide construction and adjuvating

**Figure 5:**
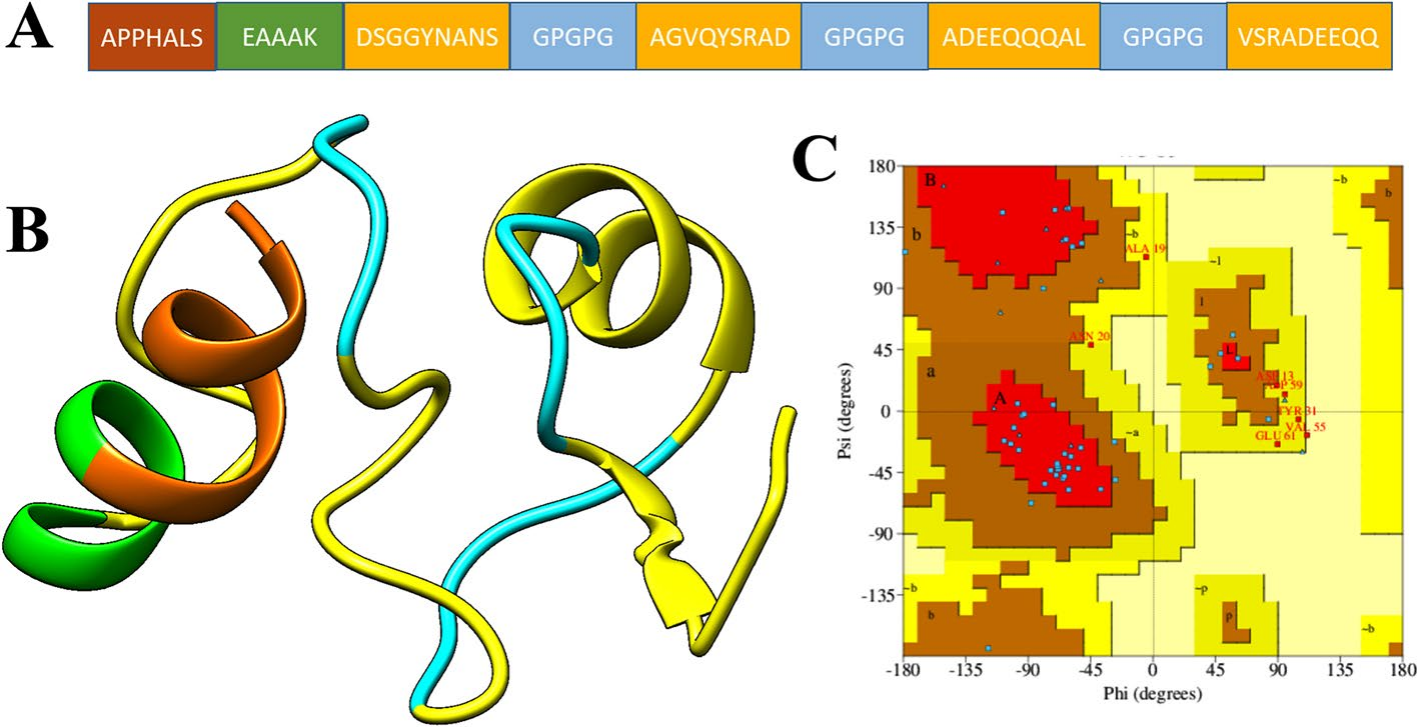
(**A**) Schematic presentation of vaccine construct composition, (**B**) 3D model of the predicted vaccine structure (yellow colors represent epitopes, brown is for adjuvant, green is for EAAAK linker, cyan is for GPGPG linker) and (**C**) Ramachandran plot the vaccine. The coloring scheme of the Ramachandran plot can be interpreted at https://www.ncbi.nlm.nih.gov/pmc/articles/PMC5734310/.

The organism and protein-based vaccines proved productive for many years in reducing mortality and preventing getting infected. However, due to extra antigenic load of these vaccines often resulted in inaccurate immune responses^15^. Peptide vaccines are a good alternative to these vaccines as they are specific in action and are more accurate as well as easy to produce with the minimum cost required^90^. Nevertheless, such peptide vaccines generate low immunogenic responses which can be overcome by fusing multiple epitopes^91^. The final set of epitopes discussed above were fused via GPGPG liner while EAAAK linker was used to join the epitope peptide to adjuvant, which was used as an adjuvant molecule. Both the used linkers are good at keeping the epitopes and adjuvant separated and allowing their efficient presentation to the human immune system. The designed multi-epitope peptide construct is shown in Fig. 5A.

### 3D structure modeling and structural analysis

The 3D model of the vaccine was generated using ab initio method as no appropriate template was available for homology modeling^24^. The total size of the vaccine is 63 amino acids. The 3D structure is shown in Fig. 5B. Ramachandran plot analysis indicated the vaccine structure contains 63.4% residues in favored regions, 19.5% in additional allowed regions, 14.6% in generously allowed regions, and 2.4% in disallowed regions (Fig. 5C). These values indicated good quality of the predicted vaccine structure. From a secondary structure point of the view, the structure contains 28.6% helices, 71.4% of beta-turn, and gamma turns. The structure was further subjected to loop modeling that reduced the percent of disallowed residues to 1.0. Refinement analysis found model 1 as a good vaccine model as it has improved Ramachandran plot distribution with more residues in the favored regions, stable energy of 0.97, and improved clash score of 23.54. The vaccine structure also has fewer rotamers compared to the original structure.

### Physiochemical properties analysis

Different physicochemical properties of the vaccine were investigated that can be helpful in the experimental examination of the vaccine^92^. Theoretical pI value of the vaccine is 4.20. This value aids in spotting and isolating the vaccine from the 2D gel. The molecular weight of the vaccine is 6.12 kDa making the vaccine an attractive candidate due to the ease of its purification. The estimated half-life of the vaccine is 4.4 h in mammalian reticulocytes, > 20 h in yeast, and > 10 h in *Escherichia coli*. The aliphatic index of the vaccine is 39.05 whereas its grand average of hydropathicity (GRAVY) score is − 1.016.

### Molecular docking between vaccine and immune receptors

**Figure 6:**
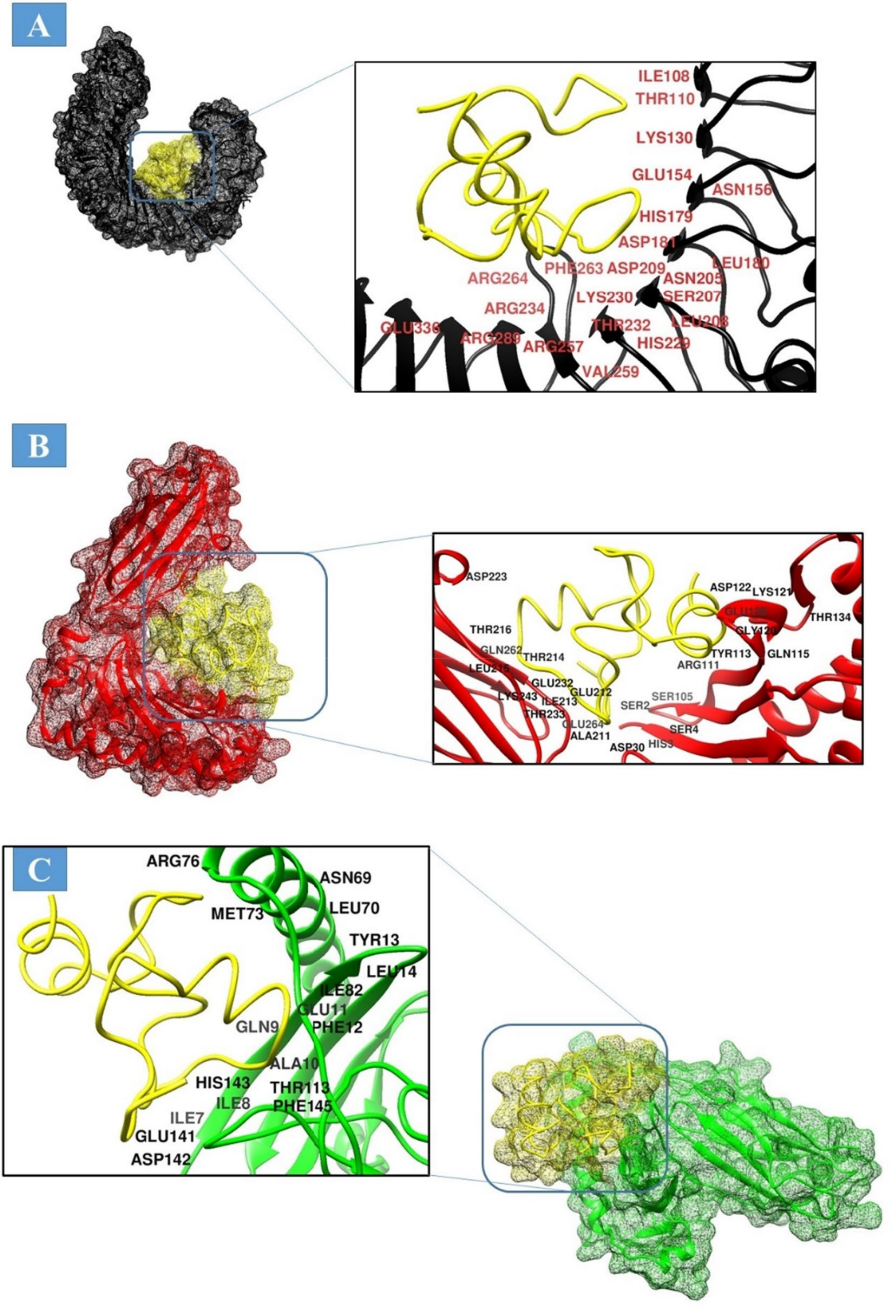
Docked conformation of the designed vaccine to different immune receptors. (**A**) TLR4 (shown in black mesh or cartoon), (**B**) MHC-I (shown in red mesh or cartoon), and (**C**) MHC-II (shown in green mesh or cartoon). The vaccine is presented as yellow mesh and stick.

Molecular docking approach was used to predict the binding mode of the designed vaccine construct with TLR4, MHC-I, and MHC-II molecules. This analysis was important in aspect to determine the presentation of the vaccine to the host immune system to activate immune signaling pathways and confer protective immunity^55^. For each receptor, 20 docked solutions were predicted which were ranked based on docking score, interacting area size of the molecules, desolvation energy, and actual rigid transformation of complexes. The complexes were then refined through FireDock webserver to remove flexibility errors. The refined solutions of PATCHDOCK are tabulated in Table 3. In case of TLR4, solution 5 was selected as the best docked complex as it has the lowest binding energy of − 10.70 kcal/mol with contribution of − 38.26 from attractive van der Waals energy, 17.92 kcal/mol from repulsive van der Waals, 19.06 from atomic contact energy (ACE) and − 3.96 from hydrogen bonding energy. TLR4 plays a significant role in inducing immune responses against *M. tuberculosis* and participates in clearing the pathogen^93^. Similarly, in case of MHC-I and II, solutions 7 and 1 were ranked as the best stable complex with net global energy of − 24.54 kcal/mol and − 43.32 kcal/mol, respectively. The binding conformation and interactions of the vaccine with different immune receptors used can be depicted in Fig. 6. For all three receptors, the vaccine acquired deep binding inside the pocket of the receptors and interact through both hydrophilic and hydrophobic bonding with several key residues of the receptors within 3 Å. In case of TLR4, these residues are; Ile108, Thr110, Lys130, Gly154, Asn156, His179, Leu180, Asp181,Asn205, Ser207, Leu208, Asp209, Lys230, His229, Thr232, Val259, Arg234, Arg257, Phe263, Arg254, Arg289 and Glu336. For vaccine and MHC-I interaction, the following residues played key role; Ser2, His3, Ser4, Asp30, Ser105, Arg111, Tyr113, Gln115, Lys121, Asp122, Gly128, Thr134, Ala211, Glu212, Ile213, Thr214, Leu215, Thr216 Thr233, Lys243, Gln262, and Glu264. In case of MHC-II, Ile7, Ile8, Gln9, Ala10, Glu11, Phe12, Leu14, Asn69, Leu70, Met73, Arg76, Ile82, Thr113, Glu141, Asp142, His143, and Phe145.

**Table 3:** Ranking of docked solutions based on different scoring functions.

Rank	Solution number	Global energy	Attractive van der Waal energy	Repulsive van der Waal energy	Atomic contact energy	Hydrogen bond energy
**TLR4**						
1	5	− 10.70	− 38.26	17.82	19.06	− 3.96
2	10	− 9.05	− 25.53	10.81	10.93	− 6.03
3	2	− 4.10	− 3.40	0.00	2.79	− 1.20
4	3	− 1.38	− 2.27	0.88	− 0.98	0.00
5	9	2.14	− 28.86	26.49	14.11	− 3.98
6	7	7.38	− 23.57	8.01	18.27	− 1.37
7	1	7.87	− 25.93	10.13	14.76	− 3.30
8	8	12.75	− 0.34	0.00	0.42	0.00
9	4	32.97	− 32.95	13.04	18.37	− 7.43
10	6	36.31	− 29.12	15.88	19.73	− 3.82
**MHC-I**						
1	7	− 24.54	− 28.62	11.86	3.52	− 3.51
2	5	− 7.58	− 10.08	7.24	6.30	− 1.28
3	2	− 5.89	− 27.24	8.74	6.18	− 2.79
4	4	6.74	− 40.96	36.24	11.19	− 3.36
5	6	11.36	− 2.78	0.00	5.37	0.00
6	1	16.72	− 6.19	2.35	2.98	− 0.46
7	9	50.16	− 13.71	61.36	10.56	− 2.54
8	8	92.25	− 36.13	98.41	14.59	− 2.78
9	3	828.31	− 28.85	1057.08	0.05	− 5.23
10	10	999.40	− 63.43	1325.32	11.17	− 986
**MHC-II**						
1	1	− 43.32	− 50.97	31.61	− 1.73	− 6.88
2	2	− 29.55	− 43.32	20.98	5.20	− 5.13
3	4	− 23.18	− 30.28	7.36	5.32	− 2.30
4	10	− 16.35	− 23.49	7.67	0.03	− 3.16
5	7	5.30	− 2.11	0.08	1.78	0.00
6	8	6.24	− 2.83	0.00	1.79	0.00
7	3	6.49	− 0.74	0.00	0.63	0.00
8	5	17.94	− 13.16	25.70	3.77	0.00
9	9	18.92	− 7.78	17.52	4.96	0.00
10	6	19.70	− 7.01	0.68	1.15	− 0.31

### Structure stability analysis

**Figure 7:**
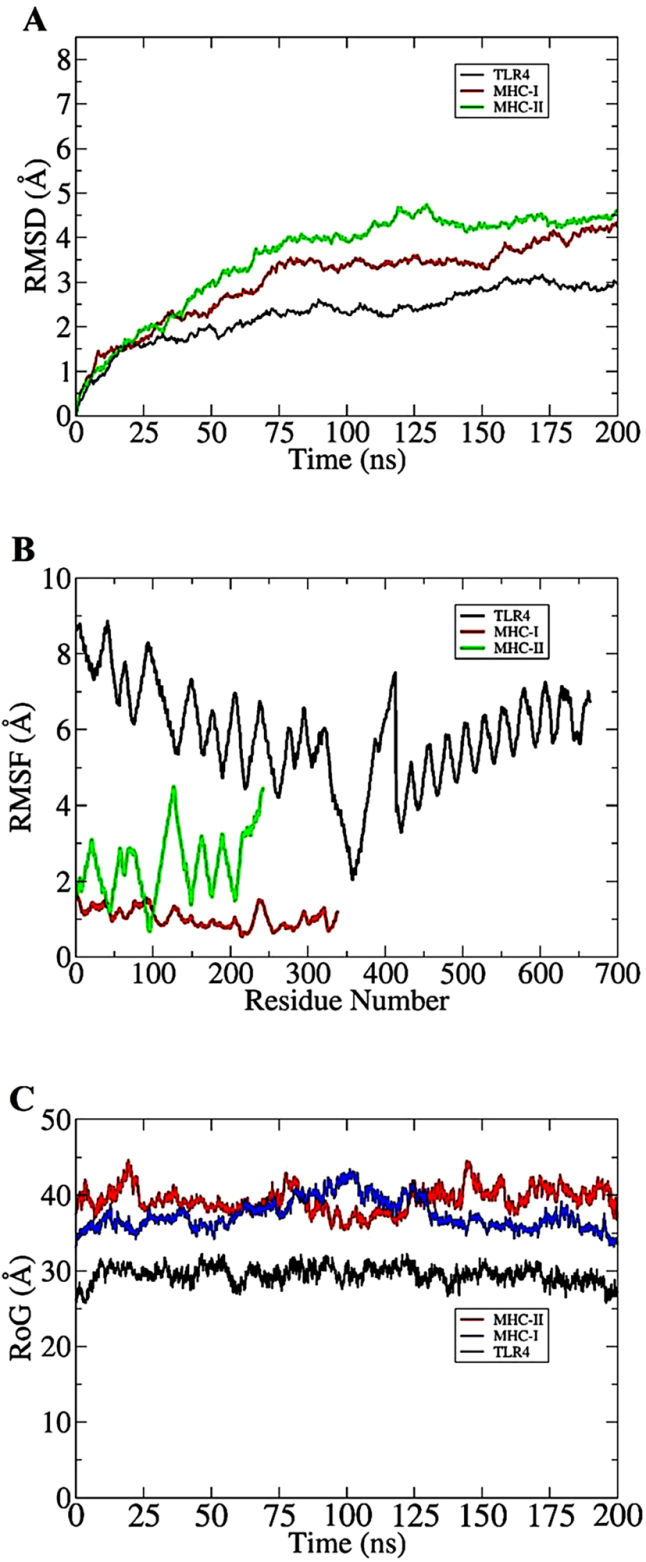
Different molecular dynamics simulation graphs. (**A**) RMSD, (**B**) RMSF, and (**C**) RoG.

The docked complexes were used further in 200 ns of molecular dynamics simulation to investigate their intermolecular interactions stability. First, carbon alpha based root mean square deviations (RMSD)^94^ of the complexes was evaluated and rendered in Fig. 7A. As can be seen, the complexes dynamics behavior is very stable with no sharp deviations observed, which indicates that docked complexes have not experienced any significant binding conformational changes and remained intact during simulation time. The RMSD of all systems is within the range of 4 Å. The vaccine-TLR4 system in particular is more stable than the other two systems with RMSD fluctuating around 2–2.5 Å. Second, the residue level fluctuations of the vaccine-receptor(s) were examined using carbon alpha root mean square fluctuations (RMSF)^95^ (Fig. 7B). As found in the RMSD, systems are quite stable and can be reflected from RMSF analysis. Both residues of the receptors and vaccine are in a good stable energy phase despite few fluctuations in the vaccine which could be due to the presence of loops because of their flexible nature. Further validation on the complex compactness and equilibrium was obtained using radius of gyration (RoG) analysis^96^ (Fig. 7C). The RoG of the systems is found between 25 Å and 45 Å. The RoG results are showing homology to that of RMSD and is validating the good stability of complexes. As indicated in RMSD analysis, systems dynamics are very uniform, and no major spike was highlighted. The TLR4 is seen in more stability than MHC-I and MHC-II.

### Binding free energy estimation

Biophysical basis of the vaccine recognition by the human immune receptors was understood by employing AMBER MMGB/PBSA method. Such energies calculations are considered more accurate than molecular docking approaches and use moderate computational power compared to extensive Alchemical perturbation methods^97^. The binding free energies of all complexes are summarized in Table 4. Among the complexes, the MHC-I complex is highly stable in both MMGBSA and MMPBSA, followed by TLR4 and MHC-II complex. All the complexes revealed highly favorable gas phase energy and both of its components (electrostatic and van der Waals) reported significant contribution. The gas phase energy of TLR4, MHC-I, and MHC-II binding energy is − 541.11 kcal/mol, − 702.05 kcal/mol, and − 425.45 kcal/mol, respectively. In the solvation part, polar solvation energy was found highly unfavorable while non-polar favorable towards net binding free energy of the systems. The net solvation energy of the systems is; TLR4 (370.67 kcal/mol in MMGBSA and 394.3 kcal/mol in MMPBSA), MHC-I (458.04 in kcal/mol in MMGBSA and 518.46 kcal/mol in MMPBSA), and MHC-II (375.18 in kcal/mol in MMGBSA and 386.94 kcal/mol in MMPBSA).

**Table 4:** Different energy components estimated in MMGBSA and MMPBSA for vaccine-receptor(s) complexes.

Energy component	TLR4		MHC-I		MHC-II	
	MMGBSA	MMPBSA	MMGBSA	MMPBSA	MMGBSA	MMPBSA
ΔE_vdW_	− 195.22	− 195.22	− 245.07	− 245.07	− 211.12	− 211.12
ΔE_ele_	− 345.89	− 345.89	− 456.98	− 456.98	− 214.33	− 214.33
Epolar.solv	400.84	422.64	487.36	545.68	420.18	428.61
Enon-polar.solv	− 30.17	− 28.34	− 29.32	− 27.22	− 45.00	− 41.67
ΔG_gas(PB/GBSA)_	− 541.11	− 541.11	− 702.05	− 702.05	− 425.45	− 425.45
ΔG_sol(PB/GBSA)_	370.67	394.3	458.04	518.46	375.18	386.94
ΔG_bind(MM/PB-GBSA)_	− 170.44	− 146.81	− 244.01	− 183.59	− 50.26	− 38.51

### Estimation of binding entropy

To estimate binding entropy for each complex, the normal mode analysis method of AMBER was employed and executed through CPPTRAJ module. The net binding entropy estimated for TLR4-vaccine, MHC-I-vaccine, and MHC-II-vaccine is 9.48 kcal/mol, 4.85 kcal/mol, and 5.23 kcal/mol, respectively. These findings suggest efficient trapping of the vaccine at the docked site of the receptors.

### Immune simulation studies

**Figure 8:**
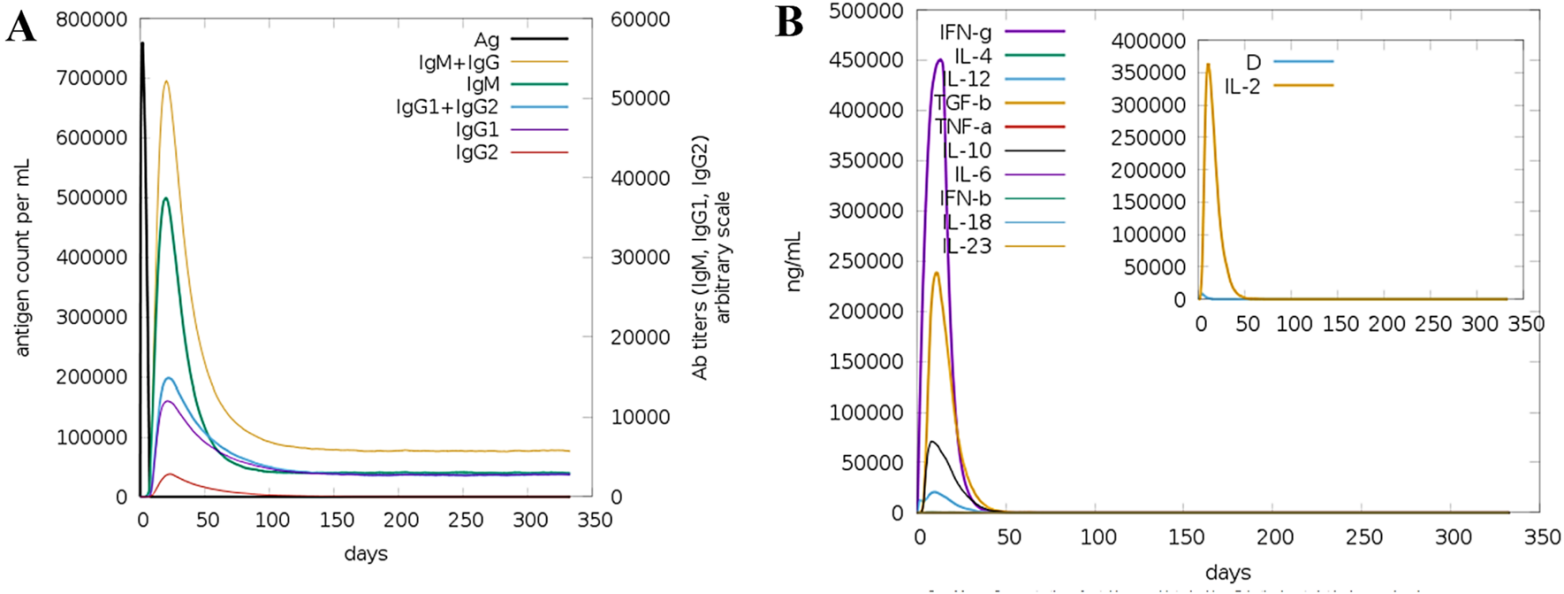
Simulated immune responses against the design vaccine antigen. (**A**) Antibodies titer count, (**B**) interleukin and interferon responses to the vaccine.

The introduction of designed vaccine construct to host body stimulates high production of secondary immune responses as a mark increase in IgM + IgG was spotted. The IgM response as primary immune response is also high. Tertiary immune reactions were also observed in the form of IgM + IgG, IgM, IgG1 + IgG2, IgG1, and IgG2. The IgG2 level is very low which might be less stimulated by the vaccine antigen thus describing its low immune protection against the pathogen. Also, a significant response of IFN-g (> 400,000 ng/ml) was also noticed. The immune responses in response to the vaccine antigen are illustrated in Fig. 8.

### Codon optimization and in silico cloning

**Figure 9:**
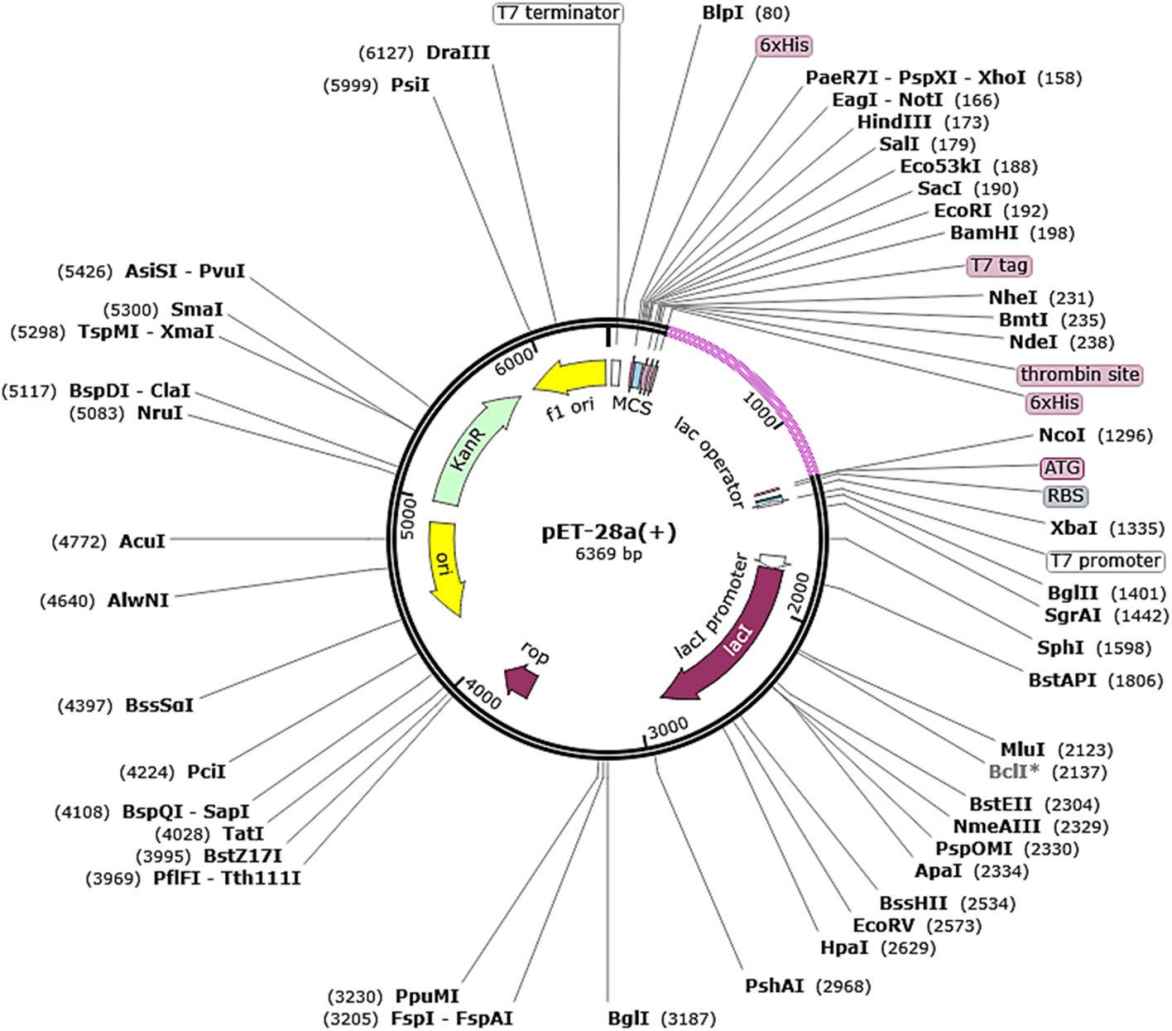
Cloned vaccine (shown in magenta color) into pET-281(+) vector.

The vaccine amino acid sequence was reverse translated to nucleotide sequence and was used in codon optimization analysis. This analysis was vital to ensure good expression of the vaccine in targeted expression system i.e. *E. coli* K12. The GC content of the improved sequence is 48.13 which is very close to that of *E. coli* K12 system (50.73). The codon adaptation index (CAI) of the vaccine is 1, demonstrating an ideal value for achieving good expression. The in silico cloned vaccine can be seen in Fig. 9.

### Disulfide engineering of the vaccine

**Figure 10:**
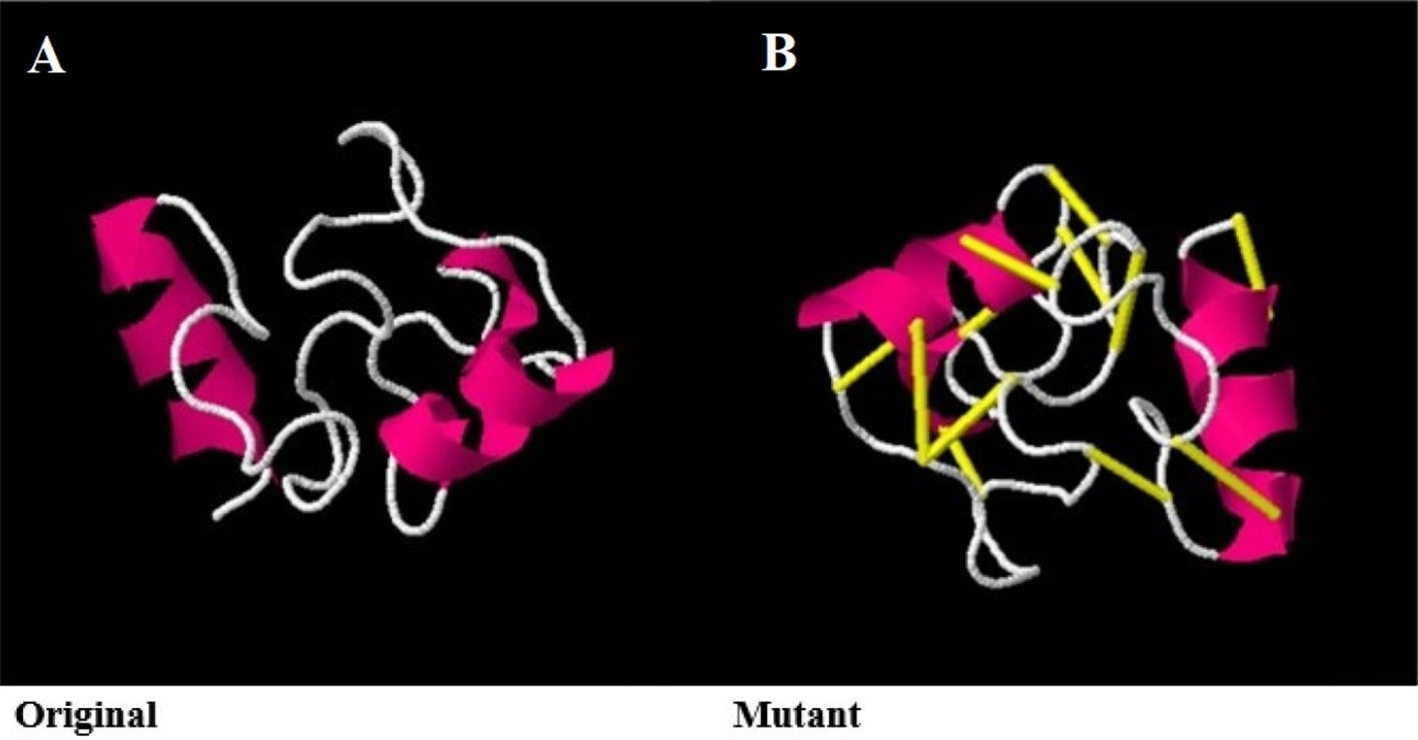
Disulfide engineering of the designed vaccine. The wild type (**A**) as well mutant structure (**B**) are presented. The yellow color stick represents introduced disulfide bonds.

Next, disulfide engineering of the vaccine construct was accomplished to get stability of molecular interactions and confer conformational geometer stability of the vaccine^98^. Total 8 residues (Ala1-His4, Pro2-Gly26, Ala5-Ala27, Lys12-Tyr17, Ser14-Tyr31, Gly22-Gln47, Pro25-Pro51, and Val29-Gly54) with a binding energy value of higher 3 kcal/mol were mutated as shown in Fig. 10. The number of pair residues of the vaccine predicted that can be mutated to render structural stability is listed in Table S3.

## Conclusions

The present work describes in silico based multi-epitope vaccine design for *M. tuberculosis* based on fully sequenced 200 genomes. The epitopes mapping was done using two potential vaccine candidates: diacylglycerol acyltransferase and ESAT-6-like proteins. The vaccine construct comprises core antigenic peptides, which are non-allergic, non-toxic, and highly water soluble. Host immune stimulation in response to the vaccine antigen proved the good production of primary, secondary, and tertiary immune responses as well as good interferon and interleukins counts. The vaccine construct is also revealed to show robust interactions with TLR4, MHC-I, and MHC-2 immune receptors and are dynamically stable. Further confirmation on the vaccine binding strength and to highlight atomic level interactions energy, binding energy estimation was performed that depicted both van der Waals and electrostatic energy dominating in complexes formation. Though we used very strict criteria for the selection of epitopes, still several points can be improved in future studies. For example, the extend of immune protection the designed vaccine ensemble will provide is not known and must be evaluated in in vivo animal models^55^. The epitopes order used in the vaccine construct also requires a great deal of experimental testing to get optimal immunogenicity responses^99^. Computational vaccine design strategies in the recent past attracted significant scientific attention because of their applicability to a range of cultured and non-cultured pathogens as well as because of straight forward nature, cost effective and need of less time^100^. Computer aided vaccine design strategies have been successfully applied in the development of Meningococcus B (MenB^101^), *Staphylococcus aureus*^102^*,* Chlamydia, *Streptococcus pneumonia,* and *group* A *Streptococcus.* In a nutshell, we proposed that the designed vaccine is ready to be utilized for further experimentations to evaluate its biological potency against drug resistant *M. tuberculosis*.

## Supplementary Information

Supplementary Figure 1.

Supplementary Figure 2.

Supplementary Table 1.

Supplementary Table 2.

Supplementary Table 3.

Supplementary Legends.
