# Supplementary figures and images for: An integrated computational framework to design a multi-epitopes vaccine against Mycobacterium tuberculosis
Source: Sci Rep. 2021 Nov 9;11:21929. doi: 10.1038/s41598-021-01283-6 (PMC8578660; doi:10.1038/s41598-021-01283-6)

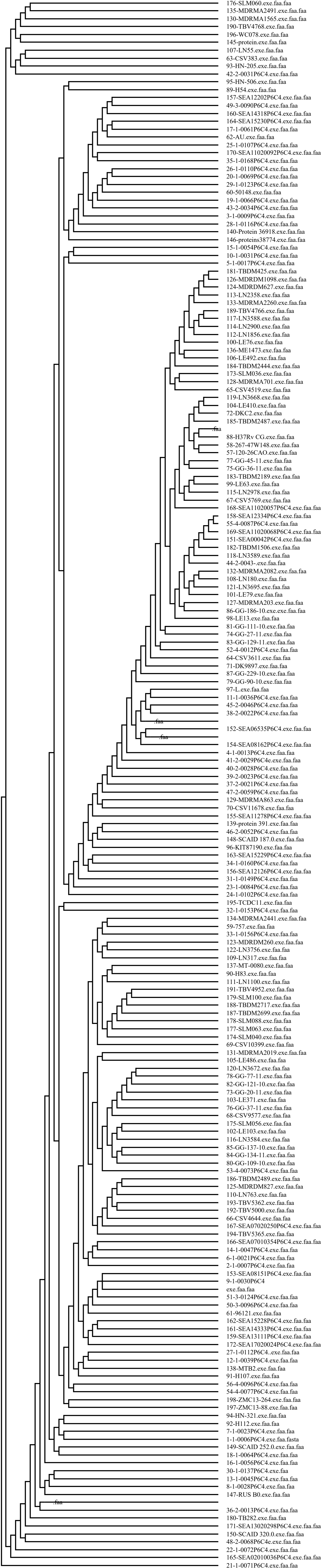

0 1.25001359210699 2.50002718421398 3.75004077632096 5.00005436842795

MYA (CORE PHYLOGENY)

Supplement: Supplementary file 1 — Supplementary Figure 1. [file 41598_2021_1283_MOESM1_ESM.pdf]

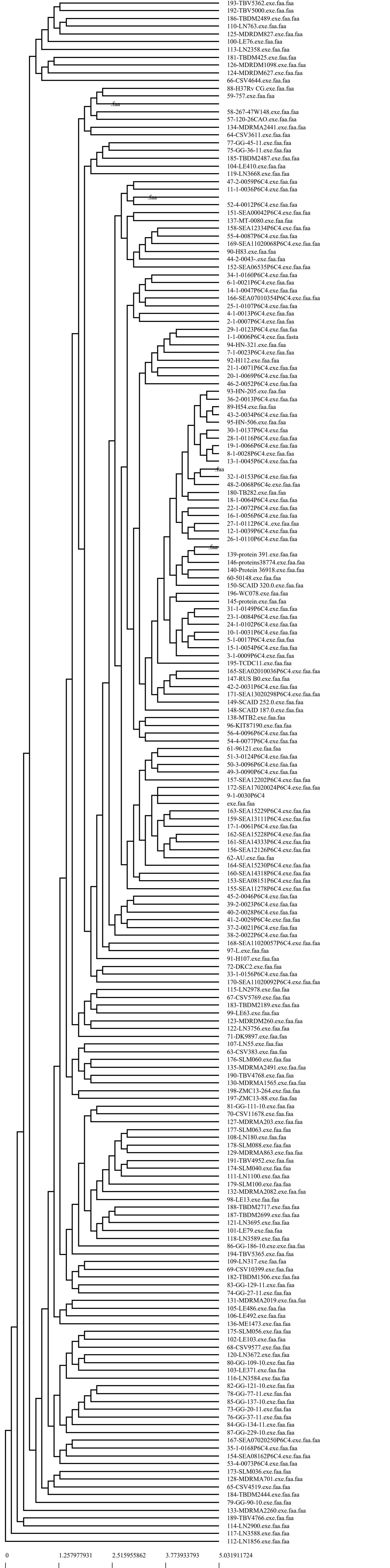

0 1.257977931 2.515955862 3.773933793 5.031911724 MYA (PAN\_PHYLOGENY)

Supplement: Supplementary file 2 — Supplementary Figure 2. [file 41598_2021_1283_MOESM2_ESM.pdf]
