# Supplementary material for: An integrated computational framework to design a multi-epitopes vaccine against Mycobacterium tuberculosis
Source: Sci Rep. 2021 Nov 9;11:21929. doi: 10.1038/s41598-021-01283-6 (PMC8578660; doi:10.1038/s41598-021-01283-6)
Supplement: Supplementary file 5 — Supplementary Table 3. [file 41598_2021_1283_MOESM5_ESM.docx]

| **Res1 Seq #** | **Res1 AA** | **Res2 Chain** | **Res2 Seq #** | **Res2 AA** | **Chi3** | **Energy** | **Sum B-Factors** |
| --- | --- | --- | --- | --- | --- | --- | --- |
| 1 | ALA |  | 4 | HIS | -95.5 | 3.1 | 0 |
| 2 | PRO |  | 26 | GLY | -61.39 | 8.09 | 0 |
| 5 | ALA |  | 27 | ALA | -88.52 | 5.34 | 0 |
| 12 | LYS |  | 17 | TYR | -110.35 | 5.22 | 0 |
| 14 | SER |  | 31 | TYR | 81.71 | 6.63 | 0 |
| 22 | GLY |  | 47 | GLN | 98.03 | 5.02 | 0 |
| 24 | GLY |  | 27 | ALA | 85.24 | 2.9 | 0 |
| 25 | PRO |  | 51 | PRO | -66.01 | 5.93 | 0 |
| 26 | GLY |  | 52 | GLY | 98.83 | 1.33 | 0 |
| 26 | GLY |  | 54 | GLY | -81.05 | 1.55 | 0 |
| 29 | VAL |  | 39 | PRO | 105.58 | 3.16 | 0 |
| 29 | VAL |  | 44 | GLU | 124.81 | 5.24 | 0 |
| 29 | VAL |  | 48 | ALA | -66.22 | 3.33 | 0 |
| 34 | ALA |  | 58 | ALA | -98.14 | 0.58 | 0 |
| 41 | ALA |  | 44 | GLU | 95.12 | 2.27 | 0 |
| 53 | PRO |  | 56 | SER | 99.17 | 2.27 | 0 |
| 57 | ARG |  | 62 | GLN | -71.03 | 3.48 | 0 |
