# Supplementary material for: An integrated computational framework to design a multi-epitopes vaccine against Mycobacterium tuberculosis
Source: Sci Rep. 2021 Nov 9;11:21929. doi: 10.1038/s41598-021-01283-6 (PMC8578660; doi:10.1038/s41598-021-01283-6)
Supplement: Supplementary file 6 — Supplementary Legends. [file 41598_2021_1283_MOESM6_ESM.docx]

**Supplementary Files Legends**

**S-Fig.1.** Core proteome based phylogenetic tree of 200 *M. tuberculosis* strains.

**S-Fig.2.** Pan proteome based phylogenetic tree of 200 *M. tuberculosis* strains.

**S-Table 1.** List of 200 strains used in pan proteome analysis for identification of core proteome.

**S-Table 2.** Number of core, accessory, unique and absent proteins in each strain of *M. tuberculosis*.

**S-Table 3.** Number of pair residues of the vaccine predicted that can be mutated to render structural stability.
